# 

**Published:** 1909-05-15

**Authors:** 


CONTENTS

Event and Comment - - - - - - - - 227

Mechanical Dentistry Forty Years Ago,
*By Edwin D. Downs, D.D.S.* 229

Gold as a Filling Material, with Special Reference to Plastic Gold,
*By C. G. Morsheimer, D.D.S.* 235

Pyorrhea Alveolaris; its Prevention and Cure,
*By Joseph W. Wassall, M.D., D.D S.* 238

A Rational Method of Producing Local Anesthesia,
*By Herman Prinz, M.D., D.D.S.* 242

Oral Surgery, - - - - - *By Dr. Wm. E. Chenery* 255

Lame Teeth, - - - - - *By R. Ottolengin, M.D.S.* 260

Obituary - - - - - - - - - - 263

Indents - - - - - - - - - - - 265

Announcements - - - - - - - - - 278

